# The ultrasound-guided proximal intercostal block: anatomical study and clinical correlation to analgesia for breast surgery

**DOI:** 10.1186/s12871-019-0762-2

**Published:** 2019-06-05

**Authors:** Nantthasorn Zinboonyahgoon, Panya Luksanapruksa, Sitha Piyaselakul, Pawinee Pangthipampai, Suphalerk Lohasammakul, Choopong Luansritisakul, Sunsanee Mali-ong, Nawaporn Sateantantikul, Theera Chueaboonchai, Kamen Vlassakov

**Affiliations:** 1 Department of Anesthesiology, Siriraj Hospital, Mahidol University, 2 Phranok road, Bangkoknoi, 10700 Thailand; 2 Department of Orthopedic Surgery Siriraj Hospital, Mahidol University, 2 Phranok road, Bangkoknoi, 10700 Thailand; 3 Department of Anatomy, Siriraj Hospital, Mahidol University, 2 Phranok road, Bangkoknoi, 10700 Thailand; 4 Department of Surgery, Siriraj Hospital, Mahidol University, 2 Phranok road, Bangkoknoi, 10700 Thailand; 5 Department of Anesthesiology, Perioperative and Pain Medicine, Brigham and Women’s Hospital, Harvard Medical School, 75 Francis Street, Boston, MA 02115 USA

**Keywords:** Nerve block, Paravertebral space, Intercostal space, Intercostal block, Breast surgery

## Abstract

**Background:**

The ultrasound-guided proximal intercostal block (PICB) is performed at the proximal intercostal space (ICS) between the internal intercostal membrane (IIM) and the endothoracic fascia/parietal pleura (EFPP) complex. Injectate spread may follow several routes and allow for multilevel trunk analgesia. The goal of this study was to examine the anatomical spread of large-volume PICB injections and its relevance to breast surgery analgesia.

**Methods:**

Fifteen two-level PICBs were performed in ten soft-embalmed cadavers. Radiographic contrast mixed with methylene blue was injected at the 2nd(15 ml) and 4th(25 ml) ICS, respectively. Fluoroscopy and dissection were performed to examine the injectate spread. Additionally, the medical records of 12 patients who had PICB for breast surgery were reviewed for documented dermatomal levels of clinical hypoesthesia. The records of twelve matched patients who had the same operations without PICB were reviewed to compare analgesia and opioid consumption.

**Results:**

Median contrast/dye spread was 4 (2–8) and 3 (2–5) vertebral segments by fluoroscopy and dissection respectively. Dissection revealed injectate spread to the adjacent paravertebral space, T3 (60%) and T5 (27%), and cranio-caudal spread along the endothoracic fascia (80%). Clinically, the median documented area of hypoesthesia was 5 (4–7) dermatomes with 100 and 92% of the injections covering adjacent T3 and T5 dermatomes, respectively. The patients with PICB had significantly lower perioperative opioid consumption and trend towards lower pain scores.

**Conclusions:**

In this anatomical study, PICB at the 2nd and 4th ICS produced lateral spread along the corresponding intercostal space, medial spread to the adjacent paravertebral/epidural space and cranio-caudal spread along the endothoracic fascial plane. Clinically, combined PICBs at the same levels resulted in consistent segmental chest wall analgesia and reduction in perioperative opioid consumption after breast surgery. The incomplete overlap between paravertebral spread in the anatomical study and area of hypoesthesia in our clinical findings, suggests that additional non-paravertebral routes of injectate distribution, such as the endothoracic fascial plane, may play important clinical role in the multi-level coverage provided by this block technique.

## Background

Regional anesthesia has been consistently associated with superior pain control, lower opioid use and related side effects, when compared to conventional opioid-based analgesia [1–3]. Applicable truncal regional techniques such as paravertebral block and intercostal block have been described [4–7]. The ultrasound-guided thoracic paravertebral block (TPVB) is considered advanced technique [8] due to relative target depth and challenging sonography window, needle visualization [9] and recognized proximity of underlying pleura and lung [10].

The intercostal space (ICS) communicates proximally (medially) with the paravertebral space - as little as 1 ml dye injected into the ICS can spread to the paravertebral space [11]. A larger-volume injection may cause further spread to the paravertebral and/or epidural space, providing multilevel analgesia with 1–2 level injections. The ultrasound-guided proximal intercostal block (PICB) is performed by injecting local anesthetics between the internal intercostal membrane (IIM) and the endothoracic fascia/parietal pleura (EFPP), closely lateral to the tip of the transverse process (TP). While the PICB has been utilized as an alternative technique to TPVB for breast anesthesia/analgesia in our institutions, the exact mechanism of the block has not been elucidated.

The goals of this study were to examine the anatomical spread of PICB injectate and explore its translation into clinical analgesia after breast surgery. The anatomy part of the study assessed the spread of methylene blue and radiographic contrast injection into the IIM-EFPPC plane of cadavers with both fluoroscopy and anatomical dissection. The clinical part consisted of a retrospective medical records review of patients who had undergone breast surgery under general anesthesia (GA) with and without PICB, examining the dermatomal analgesia/hypoesthesia distribution and the analgesic effect of the PICB.

## Methods

### Anatomy study

After IRB review and exemption, ten cadavers were prepared for the study by soft embalming technique [12]. The cadavers were legally donated to Mahidol University and the donors and their next of kin provided informed consent for the use the cadavers for academic and research purposes during the donation process, all following strictly the institutional and the national protocols and guidelines. Two anesthesiologists trained in regional anesthesia performed PICBs at the 2nd and 4th ICS under real-time ultrasound guidance (SonoSite M-Turbo, linear 38 mm 10–12 MHz transducer, Fujifilm SonoSite, Bothell, WA) and with echogenic needles (22G 50mm, Pajunk® GmbH, Geisingen, Germany The paramedian sagittal scan started by identifying the first rib, then proceeded caudally, to identify the 2nd and the 4th intercostal spaces. The ultrasound probe was then moved medially to identify the tips of the corresponding transverse processes and then moved back laterally to the proximal part of the ICS till optimal sagittal views of ribs, intercostal muscles and parietal pleura were obtained. The needle was inserted in-plane in a caudal-to-cranial direction until its tip was located under the IIM; then, anterior (downward) displacement of EFPP by the injectate provided confirmation of correct needle tip position and satisfactory injection.

**Fig. 1:**
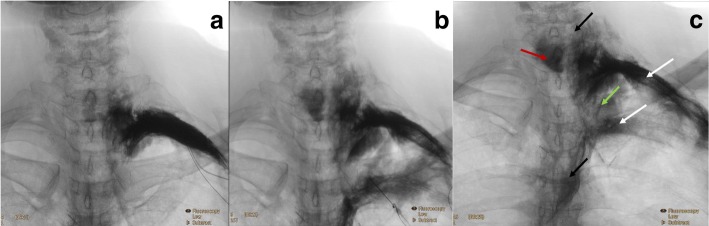
**a** Fluoroscopic image of 2nd proximal intercostal space injection; **b** Fluoroscopic image after the subsequent 4th proximal intercostal space injection; **c** The final image illustrates the distal (lateral) spread to the left 2nd and 4th intercostal spaces (white arrows), the corresponding ipsilateral paravertebral spread from C6 to T6 (black arrows), contralateral epidural spread (red arrow) and endothoracic plane spread (green arrow)

**Fig. 2:**
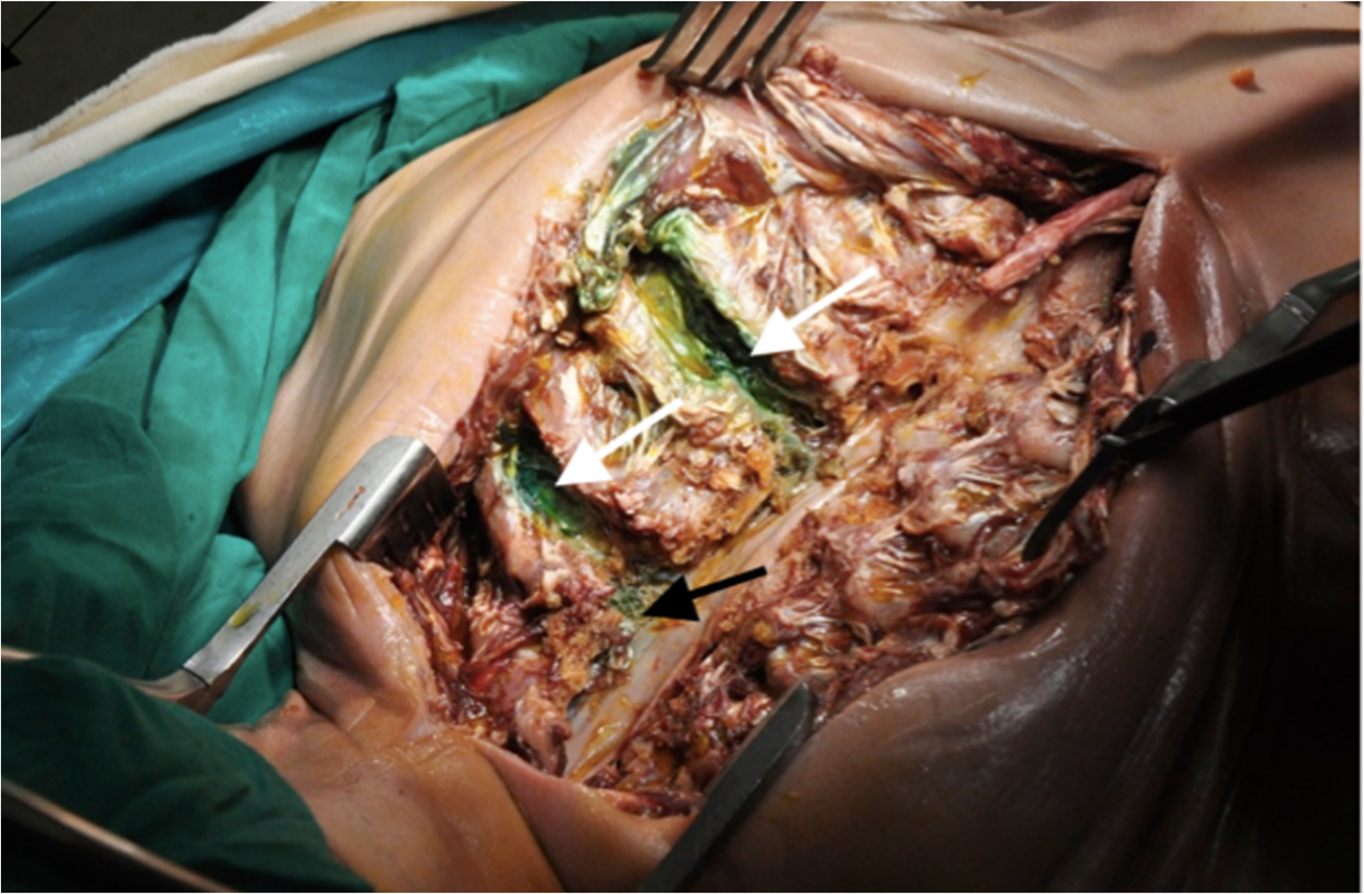
Dissection revealing 2^nd^ and 4^th^ intercostal space spread (white arrows) and paravertebral spread (black arrow)

**Fig. 3:**
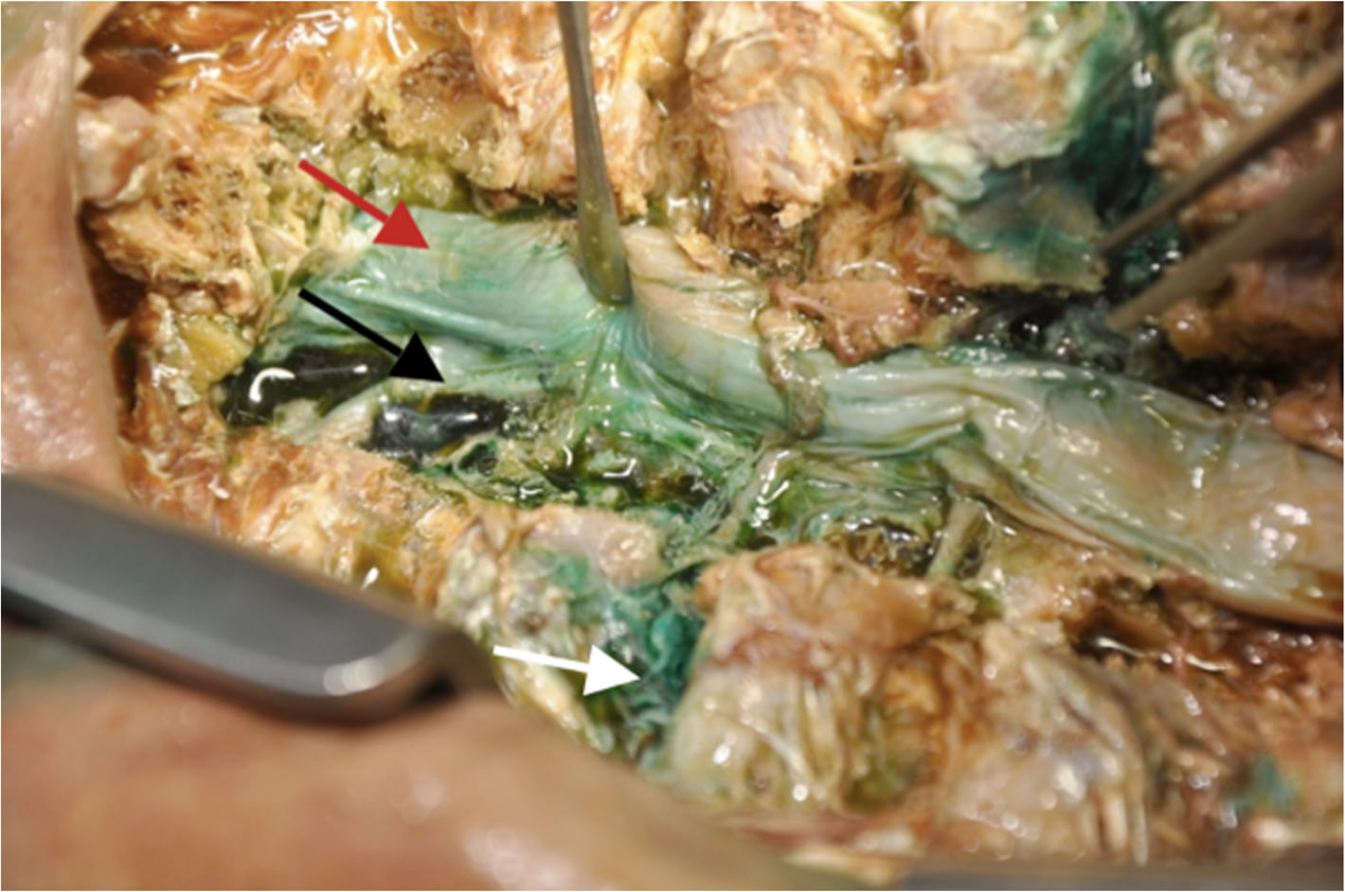
Dissection demonstrating intercostal neurovascular spread (white arrow), paravertebral spread (black arrow) and staining of the dura mater (epidural spread - red arrow)

**Fig. 4:**
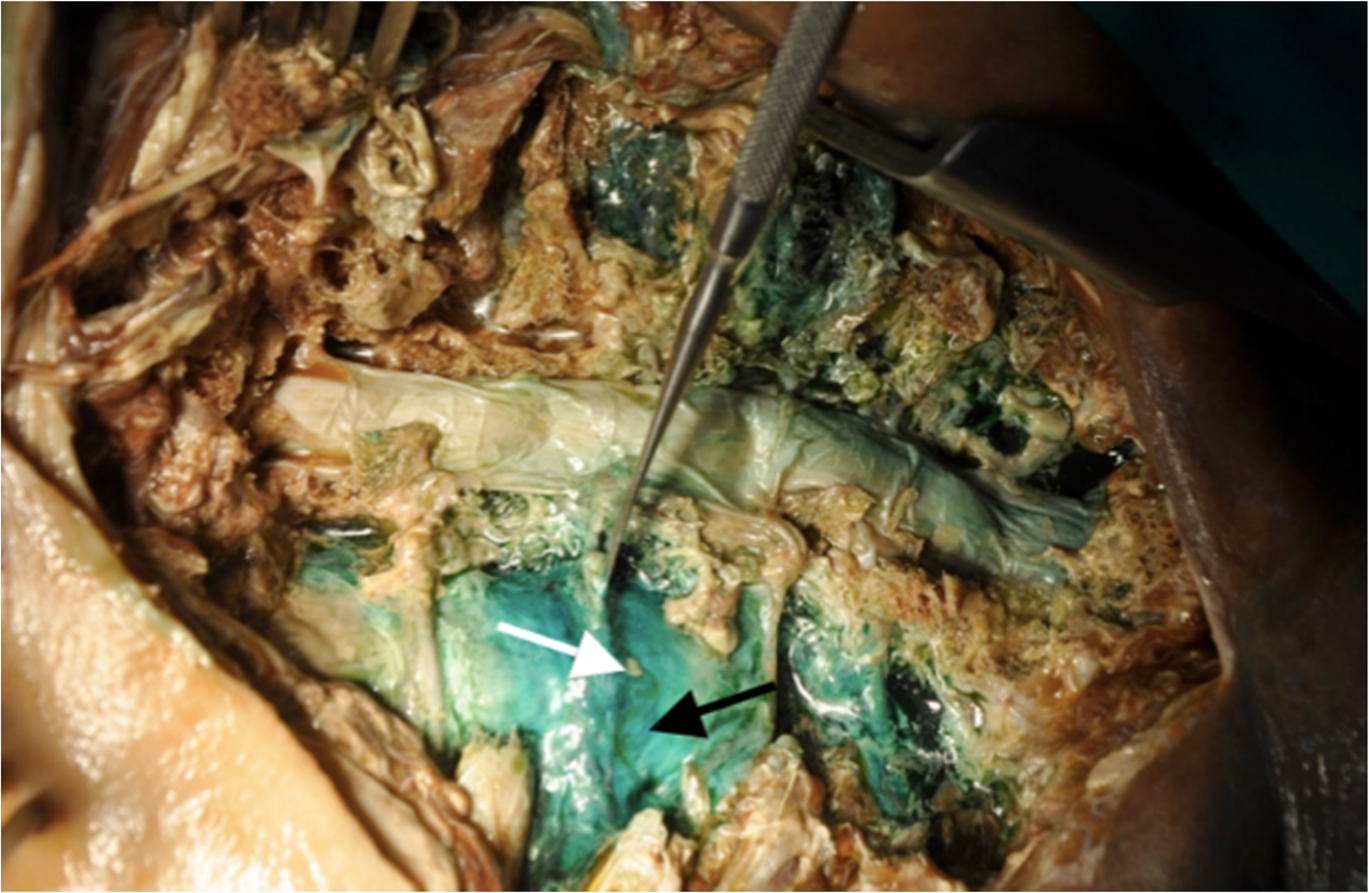
Dissection revealing trans-segmental EFPP spread (black arrow); the underlying visceral pleura showed no methylene blue staining as seen via the small opening deliberately created during the dissection (white arrow)

The injectate was prepared by mixing a radiographic contrast agent (Ultravist240; Iopromide 240 mg iodine/ml) 30 ml with methylene blue 2 ml and diluted with water to 80 ml. After the needle was in satisfactory position by ultrasound imaging, 15 ml of injectate was injected at the 2nd proximal ICS and 25 ml at the 4th proximal ICS over 1–2 min. Real-time fluoroscopy was performed and recorded immediately after each injection to evaluate the spread of contrast (Fig. 1). The cadavers were then dissected within 1 h to examine the spread of methylene blue in the intercostal, paravertebral and epidural spaces and along the endothoracic fascia plane. The dissection started from the 2nd and 4th ribs and continued towards the corresponding thoracic levels, then extended from the lower cervical spine to the mid-thoracic spine (Figs. 2, 3, 4). The interpretation of the spread of radiographic contrast [13] and methylene blue was determined in consensus by 3 clinicians (NZ, PP, PL). Challenging anatomical spread from the dissection were interpreted by an expert anatomist (SP). Significant spread to the intercostal neurovascular bundle, the paravertebral space or the epidural space was interpreted as coverage of the corresponding vertebral segment. All fluoroscopic and dissection images were deposited in an encrypted computer for subsequent review.

### Clinical study

With IRB approval, the research team identified and reviewed the medical records of 12 consecutive patients who had undergone breast surgery under general anesthesia (GA) and PICB retrospectively, in order to compare the documented dermatomal levels of analgesia and hypoesthesia with the block to the results of the anatomical study. As the PICB technique had been introduced to our institution shortly before our study, the effects of the blocks, including dermatomal spread, were being assessed and documented in great detail for quality assurance. In order to compare the analgesic effect to the GA group, we performed sample size calculations, aiming to detect a 50% decrease of pain scores in the PICB group. Kim et al. [14] showed average pain score after mastectomy to be 5/10 with SD of 2/10. Using the software tool nQuery Advisor MTT0–1 (Informer Technologies, Inc., Los Angeles, CA, USA) a sample size of 12 patients per group was calculated with alpha error of 0.05 and power of 80%.

**Fig. 5:**
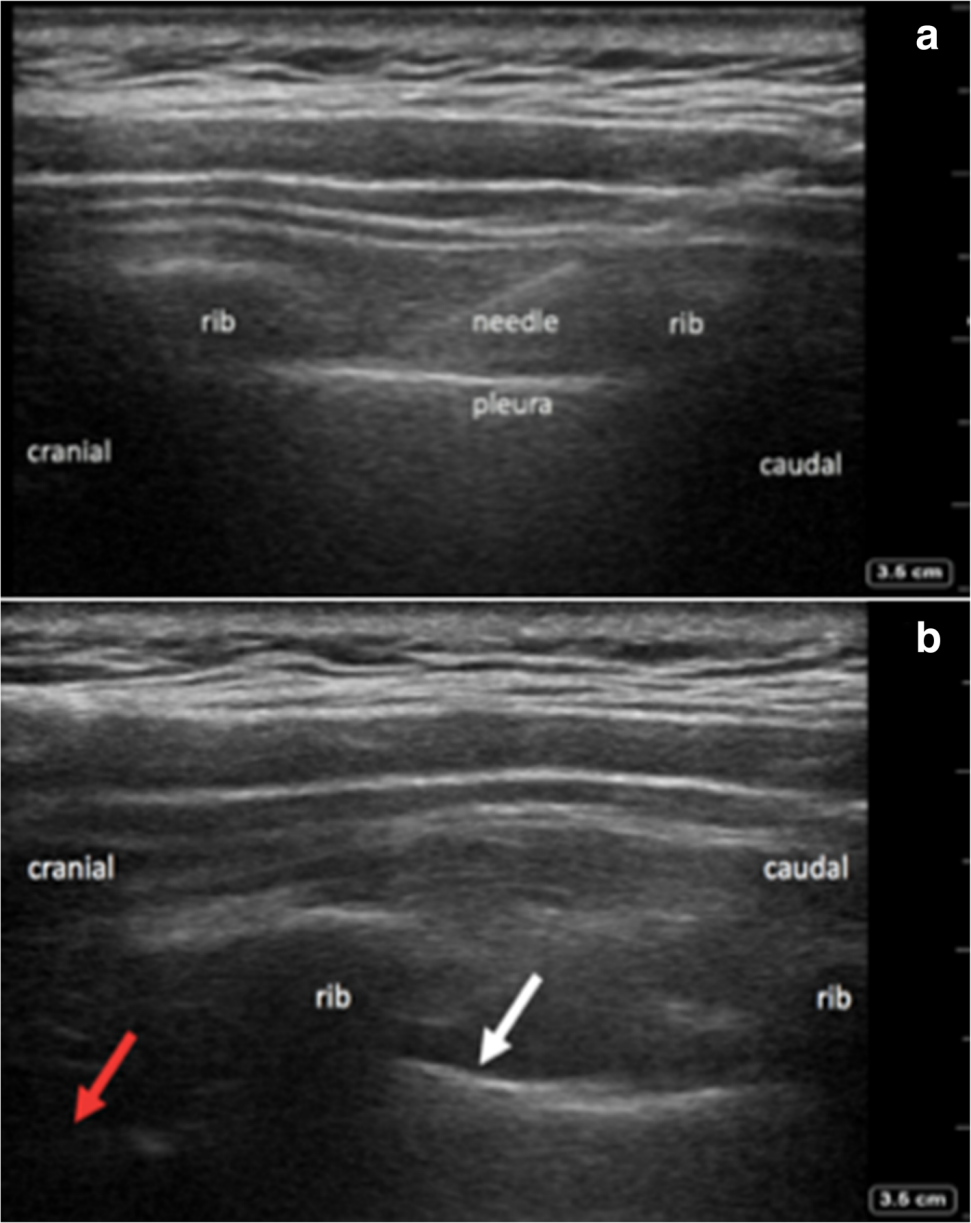
Saved ultrasound images of PICB in one of the patients from the clinical study. **a** Upper image shows the needle tip near the caudal border of the 4th rib, and just underneath the internal intercostal membrane. **b** Image below shows the anterior displacement of endothoracic fascia and parietal pleura at the level of injection (white arrow) and the next level cranially (red arrow)

The PICBs were performed using a SonoSite X-Porte US machine with a linear 38 mm 10–12 MHz ultrasound probe (Fujifilm SonoSite, Bothell, WA) and the 21G 80 mm Sonoplex needle (Pajunk® GmbH, Geisingen, Germany). Blocks were performed with standard ASA monitoring. The scanning and needling techniques were identical as in the anatomical study (Fig. 5). Once the needle was in correct position by US imaging, 10–15 ml and 20–25 ml of 0.25% bupivacaine (bupivacaine is the most affordable and most commonly used long-acting local anesthetic in Thailand), were injected into the 2nd and 4th proximal ICS, respectively (adjusted to the maximum allowable dose per body weight) to produce anterior (downward) displacement of EFPP in confirmation of optimal needle tip position and satisfactory injection.

The research team matched other 12 patients who had had the same operation with the same surgeon under general anesthesia without blocks to compare pain scores and opioids consumption. The statistical analysis included T test for normal distribution and Mann-Whitney U test for non-normal distribution, utilizing PASW statistics software (SPSS) 18.0 (SPSS Inc., Chicago, IL, USA).

All patients received general anesthesia (controlled ventilation with endotracheal tube or laryngeal mask airway). The medication choices were at the discretion of the anesthesiologist including administration of perioperative muscle relaxant, sedative and analgesics. Recorded perioperative opioid administration included all opioids given in the pre-, intra-, and post- operative periods up until discharge from the recovery room, converted to mg morphine equivalent (MME) IV units.

## Results

### Anatomy part

PICB injections were performed in 10 cadavers. Two level injections at 2nd and 4th ICS were performed in 15 chest walls. (The trial injection at other different level (T3 and T5) or TPVBs were excluded). Demographic data and injectate spread interpretation are shown in Table 1. Spinal segments coverage was assessed, separately by fluoroscopy and dissection, for an evidence of intercostal, paravertebral or/and epidural spread. As the contrast spread was interpreted with real-time fluoroscopy, whereas the anatomical dissection was performed 1 h later, discrepancies between fluoroscopic and anatomical findings could be due in part to this time gap. The median PICB coverage was 4 (range 2–8) vertebral segments by fluoroscopy and 3 (range 2–5) segments by dissection (Table 1).

**Table 1:** Demographic data of cadaver and injectate spread observed by fluoroscopy and dissection

Body	Injection number	Sex	Age (years)	Height (cm)	Side	Intercostal, paravertebral or epidural spread by fluoroscopy (segment)	Intercostal, paravertebral or epidural spread by dissection (segment)	Endothoracic spread from dissection
1	1	M	67	160	Right	C7-T6 (7)	T1-T4 (4)	yes
2	2	M	70	162	Right	T2-T6 (5)	T1-T5 (5)	yes
3	3	F	84	146	Left	C7-T6 (7)	T2-T5 (3)	yes
3	4	F	84	146	Right	T2-T5 (4)	T2-T4 (3)	yes
4	5	M	47	175	Left	T2-T5 (4)	T2, T4 (2)	yes
4	6	M	47	175	Right	T2, T4-T5 (3)	T2-T4 (3)	yes
5	7	M	77	174	Left	T2, T4 (2)	T2-T4 (3)	yes
5	8	M	77	174	Right	T2, T4 (2)	T2-T4 (3)	yes
6	9	M	57	162	Left	T2, T4-T5 (3)	T2, T4 (2)	no
6	10	M	57	162	Right	T2, T4 (2)	T2, T4 (2)	no
7	11	M	72	153	Left	T2, T4-T6 (4)	T2, T4-T6 (4)	yes
7	12	M	72	153	Right	T2-T4 (3)	T2-T4 (3)	yes
8	13	M	65	110	Left	T2, T4 (2)	T2, T4 (2)	no
9	14	NA	NA	NA	Right	C6-T6 (8)	T1, T2, T4 (3)	yes
10	15	NA	NA	NA	Left	T2-T5 (4)	T2-T5 (4)	yes

**Fig. 6:**
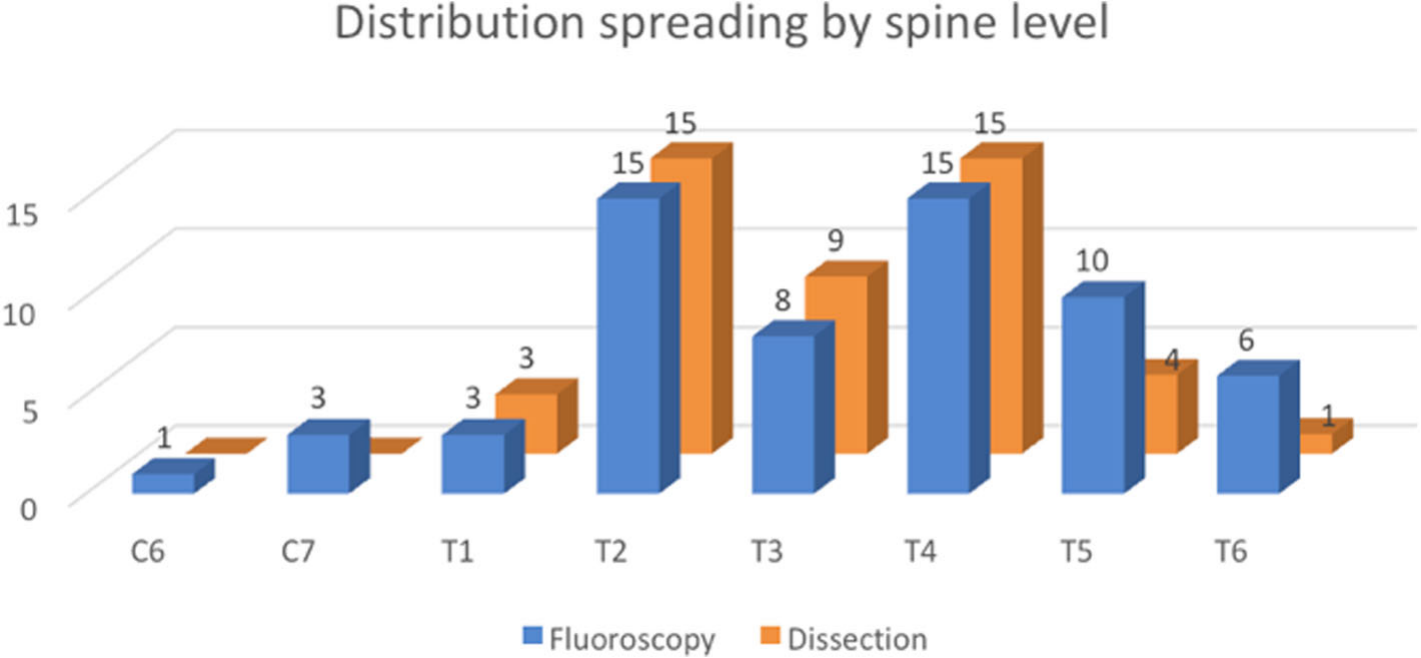
Distribution of radiographic contrast by fluoroscopy (blue) and of methylene blue by dissection (orange) from 15 two-level injections in cadavers, by spine segmental level

T2 and T4 levels were covered 100% by intercostal spread, by both fluoroscopy and dissection. However, adjacent T3 paravertebral/epidural spread was 53% (fluoroscopy) and 60% (dissection), whereas adjacent T5 level coverage was 67% (fluoroscopy) and 27% (dissection) (Fig. 6).

Eighty percent (12 of 15 specimens) of the dissections showed methylene blue staining of the endothoracic fascia at least from 2nd to 5th ICS, without any staining of the visceral pleura (Fig. 4). Three specimens revealed no endothoracic or ICS spread, but extensive paraspinal muscle staining.

The average distances from midline (spinous processes) to needle entry points were 4.35+/− 1.06 cm at the 2ndICS and 3.8+/− 1.13 cm at the 4thICS. The average depth (measured by ultrasound perpendicularly from skin to the tip of the needle in final position) was 2.01+/− 0.56 cm at the 2ndICS and 1.72+/− 0.40 cm at the 4thICS. The average needle visualization, by needle visualization score was fair. (Graded by 0 = poor needle visualization, 1 = fair needle visualization, 2 = good needle visualization. The scores were 1.00+/− 0.71 for the 2ndICS and 1.15+/− 0.80 for the 4thICS).

### Clinical part

The demographic data and the dermatomal hyposesthesia/analgesia distribution in the 12 patients who underwent breast surgeries with PICB are presented in Table 2. There were no observed and reported procedure-related complications in the patients who received PICB.

**Table 2:** Demographic data, type of operation, amount of local anesthetic and dermatomal level after proximal intercostal space block

Patient	Age (years)	BMI (kg/m^2^)	Operation	Dermatomal level	Amount of 0.25% bupivacaine (2nd/4th ICB, ml)
1	47	22	MRM	T1-T5	10/20
2	51	21	WE with SLNB	T2-T6	15/20
3	60	27	WE with needle localize	T2-T6	15/25
4	48	28	TM with SLNB	T2-T5	15/25
5	49	27	TM with ALND	T1-T6	15/25
6	53	28	TM	T1-T6	15/25
7	44	25	TM with SLNB	T1-T6	15/25
8	61	23	lumpectomy with ALND	T1-T5	10/20
9	50	17	MRM	T1-T5	10/20
10	77	27	TM with SLNB	T2-T5	15/25
11	52	32	lumpectomy with SLNB	C6-T3	15/25
12	63	28	TM with SLNB	C8-T6	15/25

*TM* total mastectomy, *MRM* modified radical mastectomy, *WE* wide excision, *SLNB* sentinel lymph node biopsy, *ALND* axillary lymph node dissection. In order to maintain the patients’ anonymity, we present BMI rounded to the nearest whole number, instead of individual weight and height in exact numbers

**Fig. 7:**
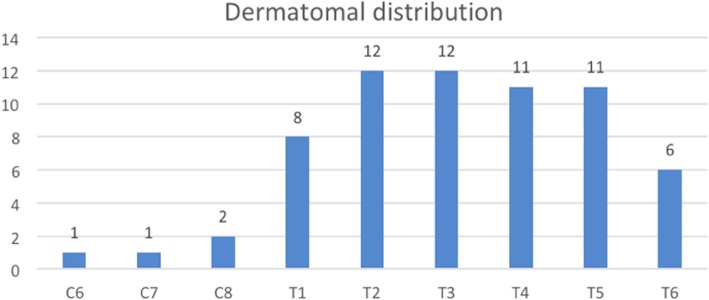
Distribution of hypoesthesia after 2th/4th PICB by dermatomal levels (12 patients)

The documented median hypoesthesia area was 5 dermatomes (range 4–7 dermatomes) and the distribution is shown in Fig. 7.

Table 3 presents demographic data of matched patients without PICB. There were no statistically significant differences in age, weight, height and BMI between the patient groups (*P* values = 0.63, 0.11, 0.57 and 0.14 respectively).

**Table 3:** Demographic data and type of operation of matched patients

Patient	Age (years)	BMI (kg/m^2^)	Operation
1	56	27	MRM
2	63	30	WE with SLNB
3	43	26	WE with needle localize
4	38	20	TM with SLNBx
5	31	35	TM with ALND
6	74	36	TM
7	68	33	TM with SLNBx
8	46	28	Lumpectomy with SLNB with ALND
9	55	25	MRM
10	64	33	TM with SLNBx
11	41	24	lumpectomy with SLNB
12	49	22	TM with SLNB with ALND

*TM* total mastectomy, *MRM* modified radical mastectomy, *WE* wide excision, *SLNB* sentinel lymph node biopsy, *ALND* axillary lymph node dissection. In order to maintain the patients’ anonymity, we present BMI rounded to the nearest whole number, instead of individual weight and height in exact numbers

The comparison of pain scores and opioid consumption between 12 patients receiving PICB and general anesthesia (GA) and 12 matched patients receiving GA alone (same operation performed by the same surgeon), is presented in Table 4.

**Table 4:** Postoperative analgesic effects of Proximal intercostal block (PICB); a comparison between PICB plus general anesthesia versus general anesthesia alone. Peri-operative opioids consumption includes opioids used during the intraoperative period and in the recovery room. Short-acting opioids include intravenous fentanyl. Long-acting opioids include intravenous morphine and meperidine

Pain scores, opioids consumption and PACU stay	GA with PICB (median/percentile; P25, P75)	GA without PICB (median/percentile; P25, P75)	*P* value
Initial numeric rating pain score in PACU (0–10)	0 (0,2.50)	0 (0,7.50)	0.671
Numeric rating pain score before discharge from PACU (0–10)	2.5 (0,3)	3.0 (2,4)	0.143
Total peri-operative opioids consumption (short and long acting opioids; intravenous morphine equivalent, mg)	7 (3.13,10.13)	11 (10,14.75)	0.004
Total peri-operative opioids consumption (long acting opioids; intravenous morphine equivalent, mg)	1 (0,2)	6 (2.50, 9.75)	0.003
PACU stay (minutes)	80 (71.25,105.00)	75 (71.25,90)	0.671

## Discussion

Truncal regional anesthesia techniques such as TPVB and the classic intercostal blocks have been utilized for anesthesia and/or analgesia for patients undergoing breast surgery [2, 4, 5]. Recent evidence also suggests that regional anesthesia techniques could potentially reduce the incidence of chronic postsurgical pain and even influence cancer recurrence [1, 15, 16]. However, TPVB is considered advanced regional anesthetic technique [8] and technically challenging due to difficulties with needle visualization [9] and identification of important collateral structures such as pleura, lung [10]. The classic intercostal nerve block is performed by landmark technique along the mid-axillary line and is considered an intermediate-difficulty technique [8]. Usually, it provides only single-dermatome analgesia per injection, therefore necessitating multiple injections to achieve analgesia for breast surgery [5]. This can be time-consuming and associated with more patient discomfort and procedural risks.

The proximal portion of the ICS (between the tip of the transverse process medially and the costal angle laterally) contains the intercostal nerves and communicates with the paravertebral space medially. Paraskeuopoulos et al. have demonstrated that as little as 1 ml methylene blue injected into the ICS 5 cm lateral to the spinous processes can spread to the paravertebral space [11]. Therefore, a larger volume PICB may result in spread into the paravertebral space and even the epidural space, providing multilevel analgesia with 1–2 level injections [17] offering alternative to TPVB.

As the breast is mainly innervated by T2-T5 spinal nerves [3] and the axilla (intercostobrachial nerve, T2) is a common site of persistent pain after axillary node dissection [18]; we utilize a combined 2nd/4th PICB technique for analgesia after breast surgery. Since pilot single-level cadaver injections demonstrated only 1–3 level spread per injection, the subsequent injections were performed with combined two-level injections, reflected in our current clinical practice. Hypothesizing that the ICSs are smaller cranially, we arbitrarily chose 15 and 25 ml for 2nd and 4th PICB, respectively. Real-time fluoroscopy demonstrated contrast consistently spreading beyond the ICS after the first 5 ml, concordant with the anatomy findings by Moorthy et al. [19] that intercostal injectate of 5 ml is confined to one ICS, whereas 10 ml spread outside the injected ICS via the potential space between the pleura and the internal intercostal muscle.

The PICBs produced consistent distribution within the injected intercostal space (100% at 2nd and 4^th^ intercostal space) but demonstrated great variability in paravertebral spread (0–7 segments), similar to the variability of paravertebral spread in TPVB described in previous studies [20, 21]. In our results, the discrepancy between paravertebral spread by anatomy dissection (60% in T3 and 27% in T5) and area of hypoesthesia in clinical finding (100% in T3 and 92% in T5 dermatome) leaves many questions. First, the sensory block area in clinical practice and the methylene blue and contrast media distribution in cadavers, may not be comparable due to different injectate viscosities and solubilities, different injection rates and pressures, and different tissue density in vivo and postmortem. Second, the ability to assess separately T3 or T5 dermatome sensation, especially when T2 and T4 dermatomes are anesthetized, is limited. Finally, while we originally hypothesized that the PICB causes multi-level analgesia through medial communication with the paravertebral space, it is plausible to consider additional non-paravertebral route(s) of distribution.

Our dissections revealed methylene blue spread inside the respective intercostal spaces and along the investing tissues around the injection sites in 80% of the specimens. The endothoracic fascia is interposed between the parietal pleura and the superior costotransverse ligament and extends laterally as an intervening fascia between pleura and internal intercostal membrane. The absence of dye on the visceral pleura and the underlying lung surface (Fig. 4) suggests that the injectate spreads above the parietal pleura and the investing layer is the endothoracic fascia. Since the confirmatory sign of a successful ultrasound-guided PICB injection is the anterior displacement of the pleura, the injectate spreads most likely in the IIM-EFPP plane. Moorthy et al. [19] demonstrated that a 10 ml of intercostal injection can cause multilevel spread (average area of spread of 51.1+/19 cm^2^) through the potential space between the pleura and the internal intercostal muscle, which supports this hypothesis. The three dissections which revealed no endothoracic or adjacent ICS spread, but extensive paraspinal muscle staining might be explained with inadvertently shallow needle placement causing injectate spread into muscle instead of endothoracic fascia plane. Predictable 2nd and 4th intercostal distribution combined with paravertebral and endothoracic fascia plane spread may present a plausible complex model for reliable dermatomal coverage of PICB in the clinical finding. The multiple anatomical routes of injectate distribution with PICB, influenced particularly by the block needle tip position relative to the internal intercostal membrane, may provide possible explanations to the inter-individual variability in segmental spread and ultimately, in clinical coverage.

Potential advantages of the PICB over TPVB (both with paramedian sagittal US scanning), include superior US-visualization of pleura and block needle due to shorter skin-to-target distance and more perpendicular US beam-to-pleura/needle orientation (unpublished data). Additionally, the longer distance of block needle from spinal canal may hypothetically convey improved safety, especially in patients who are at increased risk of bleeding complications.

Our clinical findings suggest that high-volume two-level PICBs consistently produce sensory block in dermatomes relevant to adequate analgesia after breast surgery, and could logically decrease pain and opioid consumption after mastectomy and lumpectomy. The surprisingly low median pain scores on arrival to recovery room in both groups are likely due to a combination of residual general anesthetic effect, the effect of other analgesics administered in the operating room and even individual pain thresholds. Our study was not designed and powered to examine differences in pain scores and only demonstrated a trend towards lower pain scores in the PICB group. As the shortcomings of our clinical study stem from its retrospective design with no anesthetic/analgesic standardization, well-controlled prospective trials are needed to further evaluate the analgesic, anesthetic and recovery profiles of PICB.

The discrepancy between the observed segmental spread by fluoroscopy (2–8 vertebral segments) and dissection (2–5 vertebral segments) may also seem surprising. Among the logical explanations, two appear most plausible: [1] while the contrast spread was interpreted with real-time fluoroscopy, the anatomical dissections were performed 1 h later, therefore discrepancies between fluoroscopic and anatomical findings could be due in part to this time gap; [2] it is also possible that some of the contrast spread in the paraspinous musculature could have been overinterpreted by antero-posterior fluoroscopy as “clinically useful” distribution in the paravertebral, intercostal and endothoracic fascia planes.

## Conclusions

Large-volume ultrasound-guided proximal intercostal blocks, performed at the 2nd and 4th intercostal spaces, produced a predictable lateral injectate spread along the corresponding intercostal neurovascular bundle, a less consistent medial spread to the adjacent paravertebral/epidural spaces and a contiguous endothoracic fascia plane distribution in the anatomy study. The incomplete overlap of anatomical paravertebral spread and dermatomal distribution of clinical hypoesthesia suggests additional non-paravertebral route of injectate spread, including the endothoracic fascia plane, confirmed by the staining patterns in the anatomy specimens.

## Abbreviations

ASA: American Society of Anesthesiologists
BMI: Body Mass Index
EFPP: Endothoracic fascia/parietal pleura
GA: General anesthesia
ICS: Intercostal space
IIM: Internal intercostal membrane
PICB: Ultrasound-guided proximal intercostal block
T: Thoracic (referring to vertebral/segmental or dermatomal level)
TP: Transverse process
TPVB: Ultrasound-guided thoracic paravertebral block
